# Giant Lipoma of Posterior Cervical Region

**DOI:** 10.1155/2014/289383

**Published:** 2014-10-02

**Authors:** Mahendra Singh, Ashish Saxena, Lovekesh Kumar, Snehal K. Karande, Yuvraj Kolhe

**Affiliations:** ^1^Department of Surgery, AIIMS, Jodhpur 342005, India; ^2^AIIMS Residential Complex, Basni, Jodhpur 342005, India; ^3^Department of Surgery, Hindu Rao Hospital, New Delhi 110007, India

## Abstract

Lipomas are the slow growing soft tissue tumors of benign nature. They commonly grow on torso and extremities but may also develop in head and neck region. Rarely lipomas can grow to acquire gigantic proportions, turning into an entity termed as giant lipoma. Such lipomas are entitled to immediate attention as they have a relatively high malignant potential. We report a rare case of giant cervical lipoma in an elderly gentleman, followed by a brief discussion on diagnosis and management of the disorder.

## 1. Introduction

Lipoma is a slow growing benign tumor of adipose tissue and can occur anywhere in the body. Hence, it is also known as universal or ubiquitous tumor [1]. Usually, lipomas develop on the extremities and trunk. However 13% of lipomas grow in the head and neck region [2]. Lipomas are usually small solitary lesions and rarely grow to an exceptionally large size. A lipoma is considered giant when it is greater than 10 cm in any dimension or weighs more than 1000 gm [3]. We hereby report a rare case of giant posterior cervical lipoma which was around 38 cm in greatest dimension. This appears to be by far the largest lipoma yet reported in the head and neck region, to the best of our knowledge.

## 2. Case Report

**Figure 1:**
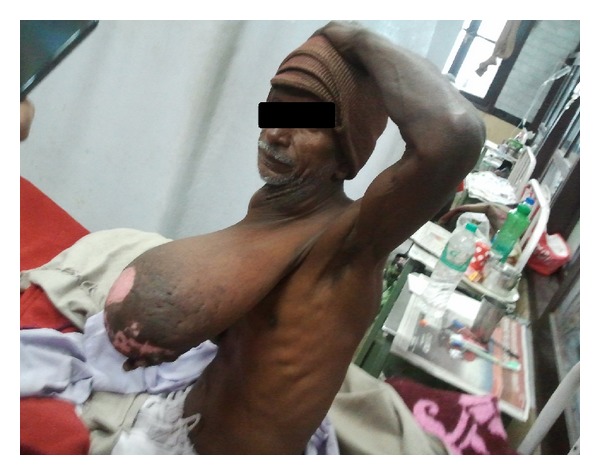
Preoperative view of lipoma.

A 62-years-old gentleman reported in surgical OPD with a giant lump hanging from the left side of neck. The swelling was present for the past 20 years. It was insidious in onset and was of small size when he noticed it first. It gradually increased in size to attain the present gigantic proportions. There was no history of pain and sudden change in size of swelling. There was not any history of fever, loss of appetite, loss of weight, difficulty in swallowing, difficulty in breathing, upper limb weakness, or voice change. Personal and family histories were unremarkable. General and systemic examinations were unremarkable. Upon local examination, a huge lump was evident hanging upon anterior aspect of the left side of chest. It was approximately 38 cm × 18 cm × 15 cm in dimensions and was extending from the left ramus of mandible to the left hypochondrium (Figure 1). Its root was attached to the posterior triangle of neck and from here the mass was hanging freely. The surface appeared to be lobulated. The overlying skin was normal except for an irregular desquamated area of size 8 cm × 5 cm in the distal most portion of lump. Serous fluid was oozing from this desquamated patch. Upon palpation the lump was neither warm nor tender. It was soft in consistency. The surface was lobulated. The lump was not fixed to the overlying skin and surrounding structures. Left carotid pulsations were normally felt. Per oral examination was essentially within normal limits. There was no regional lymph node enlargement. Sensory and motor examination of left upper limb was normal. A provisional diagnosis of lipoma was made.

**Figure 2:**
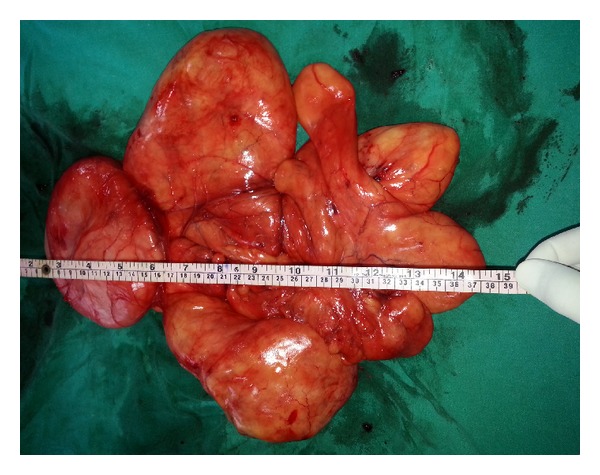
Lipoma after complete excision.

Routine investigations were unremarkable. FNAC from the swelling was suggestive of a benign fibrolipoma. A contrast enhanced CT scan of neck and thorax region was obtained to rule out any suspicious foci of malignant transformation in the mass. The mass however turned out to be essentially benign and showed no infiltration of surrounding vital structures in the neck. The patient was taken up for surgery after proper preanesthetic checkup. The mass was excised completely through an elliptical incision given below the left mandibular ramus and sent for histopathological examination (Figure 2). Postoperative period was uneventful. Histopathology report confirmed the FNAC finding of fibrolipoma. The patient was doing well until his last follow-up visit.

## 3. Discussion

Lipomas are typically slow growing tumors and only a few grow into the massive lump known as giant lipoma [3]. Mechanism behind such gigantic growth is unclear and is a matter of debate. A few studies have postulated the role of trauma, suggesting that blunt trauma can cause rupture of the fibrous septa and anchorage connections between the skin and deep fascia, allowing the adipose tissue to proliferate rapidly. One theory suggests that trauma-related fat herniation through tissue planes creates so-called pseudolipomas. It has also been suggested that trauma-induced cytokine release triggers pre-adipocyte differentiation and maturation [4]. Regardless of the mechanism, the main concern while dealing with a giant lipoma is to rule out malignancy. Liposarcoma is the most common soft tissue malignancy in the long standing lipomas; however, such occurrence is very rare. Histopathological features of dedifferentiation are the hallmark of malignant change in a benign lipoma. Dedifferentiated liposarcomas occur most frequently in the sites in which there is a chance of delayed diagnosis such as retroperitoneum. Therefore malignant changes in giant lipomas are most commonly encountered in the retroperitoneum [5]. Benign or malignant nature of a lipomatous lesion has to be established by various investigations such as ultrasonography, CT scan, MRI scan, and fine needle aspiration cytology [2, 5]. In our case, benign nature of the lipoma was confirmed by FNAC and contrast enhanced CT scan of neck and thorax.

The treatment of choice for the lipoma remains open surgical excision [6]. It is a relatively straight forward procedure, considering the encapsulated nature of lipoma. Blunt dissection along with an optimal hemostasis usually serves the purpose and preserves the surrounding structures as well. In the end, a careful histopathological examination of the excised specimen is necessary to rule out malignancy and to ensure the longevity of patient [6].

## References

[1] Davis C, Gruhn JG (1967). Giant lipoma of the thigh. *Archives of Surgery*.

[2] El-Monem MHA, Gaafar AH, Magdy EA (2006). Lipomas of the head and neck: presentation variability and diagnostic work-up. *Journal of Laryngology and Otology*.

[3] Copcu E, Sivrioglu N (2005). Posterior cervical giant lipomas. *Plastic and Reconstructive Surgery*.

[4] Signorini M, Campiglio GL (1998). Posttraumatic lipomas: where do they really come from?. *Plastic and Reconstructive Surgery*.

[5] Mnif L, Amouri A, Masmoudi MA (2009). Giant lipoma of the transverse colon: a case report and review of the literature. *Tunisie Medicale*.

[6] Allen B, Rader C, Babigian A (2007). Giant lipomas of the upper extremity. *Canadian Journal of Plastic Surgery*.

